# Loss of the vitamin D receptor in human breast and prostate cancers strongly induces cell apoptosis through downregulation of Wnt/β-catenin signaling

**DOI:** 10.1038/boneres.2017.23

**Published:** 2017-09-05

**Authors:** Yu Zheng, Trupti Trivedi, Ruby CY Lin, Colette Fong-Yee, Rick Nolte, Jeline Manibo, Yunzhao Chen, Musharraf Hossain, Konstantin Horas, Colin Dunstan, Hong Zhou, Markus J Seibel

**Affiliations:** 1 Bone Research Program, ANZAC Research Institute, Sydney, New South Wales, Australia; 2 Asbestos Diseases Research Institute, Cardiothoracic Genomics, Sydney, New South Wales, Australia; 3 School of Medical Sciences, University of New South Wales, Sydney, New South Wales 2052, Australia; 4 Department of Pathology, Shihezi University School of Medicine, Shihezi, China; 5 Biomedical Engineering, AMME, University of Sydney, Sydney, New South Wales, Australia; 6 Concord Clinical School, The University of Sydney, Sydney, New South Wales, Australia

## Abstract

Vitamin D co-regulates cell proliferation, differentiation and apoptosis in numerous tissues, including cancers. The known anti-proliferative and pro-apoptotic actions of the active metabolite of vitamin D, 1,25-dihydroxy-vitamin D [1,25(OH)_2_D] are mediated through binding to the vitamin D receptor (VDR). Here, we report on the unexpected finding that stable knockdown of VDR expression in the human breast and prostate cancer cell lines, MDA-MB-231 and PC3, strongly induces cell apoptosis and inhibits cell proliferation *in vitro.* Implantation of these VDR knockdown cells into the mammary fat pad (MDA-MB-231), subcutaneously (PC3) or intra-tibially (both cell lines) in immune-incompetent nude mice resulted in reduced tumor growth associated with increased apoptosis and reduced cell proliferation compared with controls. These growth-retarding effects of VDR knockdown occur in the presence and absence of vitamin D and are independent of whether cells were grown in bone or soft tissues. Transcriptome analysis of VDR knockdown and non-target control cell lines demonstrated that loss of the VDR was associated with significant attenuation in the Wnt/β-catenin signaling pathway. In particular, cytoplasmic and nuclear β-catenin protein levels were reduced with a corresponding downregulation of downstream genes such as Axin2, Cyclin D1, interleukin-6 (IL-6), and IL-8. Stabilization of β-catenin using the GSK-3β inhibitor BIO partly reversed the growth-retarding effects of VDR knockdown. Our results indicate that the unliganded VDR possesses hitherto unknown functions to promote breast and prostate cancer growth, which appear to be operational not only within but also outside the bone environment. These novel functions contrast with the known anti-proliferative nuclear actions of the liganded VDR and may represent targets for new diagnostic and therapeutic approaches in breast and prostate cancer.

## Introduction

Breast and prostate cancers are among the most prevalent malignancies in industrialized countries. Although mortality has steadily declined over the past 20 years, a significant proportion of patients eventually develop metastatic disease, most frequently to the skeleton.^1^ Skeletal related events due to bone metastasis are frequent and a major cause of mortality and morbidity.^2,3^

We have previously shown in rodent models that reduced bone turnover inhibits, while increased bone turnover accelerates, breast and prostate cancer growth in bone.^4–10^ These experimental findings provide a logical explanation for the clinical observation that accelerated bone turnover is associated with higher rates of skeletal related events and poorer prognosis in patients with breast or prostate cancer.^11^ They also offer a rationale for the use of anti-resorptive agents in these patients.^12^ We have further reported that in rodent models, vitamin D deficiency promotes the growth of breast and prostate cancer cells in bone.^5,8,10,13^ These effects appear to be mediated mainly through an increase in bone remodeling, that is, indirectly through changes in the bone microenvironment. However, inhibition of bone remodeling with potent anti-resorptive treatments (for example, osteoprotegerin, zoledronic acid) fails to completely reverse the pro-proliferative effects of vitamin D deficiency,^8,10^ suggesting that vitamin D deficiency may also promote cancer cell growth by an additional and possibly direct mechanism.

Apart from its function in calcium and phosphate homeostasis, vitamin D is known to exert strong anti-proliferative, pro-differentiation and pro-apoptotic actions in numerous cell types and tissues, including cancers.^14^ The biologically active metabolite of vitamin D, 1,25-dihydroxy-vitamin D [1,25(OH)_2_D], acts through binding to the vitamin D receptor (VDR), a member of the nuclear steroid hormone receptor superfamily. In the absence of vitamin D, the VDR remains in the cytoplasm. Ligand binding causes the VDR to form a heterodimer with the retinoid X receptor, which facilitates movement of the VDR-ligand complex out of the cytoplasm and into the nucleus. Within the nucleus this complex binds to vitamin D-responsive elements in the regulatory region of target genes.^15^ In keeping with the classical function of vitamin D in regulating calcium and phosphate homeostasis, the VDR is expressed at high levels in tissues such as the intestine, bone and kidney.^15^ However, research into the pleiotropic actions of vitamin D has revealed that the VDR is also expressed in numerous other tissues, including malignant tumors.^14^ Limited clinical studies in patients with breast and prostate cancer demonstrated that VDR expression in these tumors is negatively associated with tumor size and lymph node involvement.^16–18^ Furthermore, mice with global VDR knock-out show increased sensitivity to carcinogen challenges.^14,19,20^ These and the findings of our previous studies^8,10^ point to a broader role of the VDR in the regulation of cell growth, which may go beyond its classical function as a ligand-specific nuclear receptor. Using a VDR knockdown approach, the current *in vitro* and *in vivo* studies aimed to further define the role of the VDR in the regulation of breast and prostate cancer growth.

## Materials and methods

### Cell culture

The human breast cancer cell line, MDA-MB-231,^21^ was obtained from ATCC. Cells were cultured in Dulbecco's Modified Eagle's medium, supplemented with 10% fetal bovine serum (FBS, JRH Biosciences, KS, USA) and 1% penicillin-streptomycin. The human prostate cancer cell line, PC3, was obtained from ATCC. Cells were cultured in RPMI, supplemented with 10% fetal bovine serum (FBS, JRH Biosciences, Lenexa, Kansas, USA) and 1% penicillin-streptomycin solution. Unless otherwise stated, tissue culture media and supplements were from Life Technologies (Carlsbad, CA, USA).

### Knockdown of VDR expression in cancer cells

VDR expression was silenced in MDA-MB-231 and PC3 cells via a lentiviral-based expression system driving the production of short hairpin RNA species (shRNAs, Sigma, St. Louis, MO, USA). The clones selected were VDR (referred as “VDR-KD”, transfected with shRNA, TRCN000019506, Sigma) and non-target control (referred as “NT”, transfected with non-target RNA, SHC002V, Sigma). VDR-KD and NT cells from both MDA-MB-231 and PC3 cells were selected using 2 μg·mL^−1^ puromycin (Sigma, USA) for two weeks. The cells resulting from this transfection will be referred to as MDA-NT, MDA-VDR-KD, PC3-NT, and PC3-VDR-KD.

### Cell growth assays

NT and VDR-KD cells were treated with 10^-8^ mol·L^−1^ 1,25-dihydroxy vitamin D_3_ [1,25(OH)_2_D_3_] or vehicle (0.1% ethanol) in medium supplemented with 2% charcoal stripped heat-inactivated fetal bovine serum (FBS). In our hands, untreated FBS contains 1,25(OH)_2_D at a concentration of 360 pmol·L^−1^ when measured by RIA (Diasorin, Stillwater, MN, USA). In contrast, 1,25(OH)_2_D is undetectable in media supplemented with 2% charcoal stripped heat-inactivated FBS. We therefore consider culture media supplemented with 2% charcoal stripped heat-inactivated serum to be free of 1,25(OH)_2_D (“ligand-free media”). Cell growth assays were performed as described previously.^6,22^ Briefly, cells were seeded in media containing 2% charcoal stripped FBS and treated with either 10^−8^ mol·L^−1^, 1,25(OH)_2_D_3_ or vehicle. For the rescue experiments, cells were treated in the same manner with 1 μmol·L^−1^ 6-bromoindirubin-3’–oxime (BIO, Sigma), a compound that binds to, and inactivates the enzyme that normally phosphorylates and degrades β-catenin (that is, GSK-3β). Addition of BIO to the cell culture inhibits this process and therefore stabilizes β-catenin signaling,^23^ allowing for β-catenin to translocate to the nucleus and maintain Wnt/β-catenin signaling in VDR-KD cells. Media were replaced every 24 h and cells were counted daily by trypan blue exclusion until reaching 100% confluence on day four. Experiments were repeated three times in independent settings to ensure validity of the results.

### Cell apoptosis assay

NT and VDR-KD cells were cultured in medium containing 2% charcoal stripped FBS and treated with either 0.1% ethanol or 10^−8^ mol·L^−1^ 1,25(OH)_2_D_3_ for 6 days as described above. For the rescue experiment, cells were treated with vehicle or 1 μmol·L^−1^ BIO for 24 h, allowing for stabilization of β-catenin in VDR-KD cells. Apoptosis was measured by TUNEL assay using an *in situ* cell death detection kit (POD, Roche Pharmaceutical, Manheim, Germany). TUNEL-positive and total cells were counted in 3-5 random fields/well at 400X magnification.

### Transcriptome analysis and Quantitative RT-PCR

Total RNA was extracted from MDA-NT (*n*=3) and MDA-VDR-KD (*n*=3) cell cultures and was labeled and hybridized onto Affymetrix Human GeneChip Gene 2.1 ST arrays (Affymetrix, Santa Clara, CA, USA), according to the manufacturer’s instructions (Ramaciotti Centre for Genomics, University of New South Wales). Interrogating probes were imported and normalized using RMA algorithm and RMA background corrected. One-way ANOVA was carried out to extract significantly differentially expressed genes at unadjusted *P*<0.05 level. Principle component analysis indicated that these cell lines had distinctive gene expression profiles (data not shown). Pathway enrichment analysis was then carried out on the top 150 differentially expressed genes to characterize and compare the biological pathways best represented in both cell types and affected by VDR-KD. The genes are ordered by their *P-*values and based on the Kyoto Encyclopedia of Genes and Genomes (KEGG). These genes are compared to the database which consists of hundreds of carefully constructed, publicly available and curated pathway networks reflecting accumulated biological data from the field. Using Fisher’s Exact test at *P*<0.01, these top 150 genes were embedded and compared within the network structure. Accordingly, under the null hypothesis, Fisher’s Exact test was used to establish whether or not the candidate genes are associated with the pathway. The higher the enrichment score the more enriched the genes are within a specific pathway. This enabled us to infer a VDR-KD effect at the systems level^24^ and identify specifically which essential regulatory pathways are affected. To further validate the array data, cDNA was generated from the same RNA samples using Super Script III (Life Technologies). Expression levels of candidate genes were assessed by real-time RT–PCR. Primer sequences are listed in Table 1. GADPH was used for normalization.

### Western blot analysis

For protein expression, cells were harvested at 24 h post plating. Whole cell lysates were prepared using RIPA buffer and cell fractions were prepared using NE-PER Nuclear and Cytoplasmic Extraction kit (Thermo Scientific, Rockford, IL, USA), supplemented with protease inhibitor cocktail (Roche Pharmaceutical). Protein concentrations were determined using a Pierce 660 nm protein assay (Thermo Scientific), proteins were separated on Bolt 4%–12% mini gels and then transferred onto nitrocellulose membrane by means of the iBlot2 rapid transfer (Life Technologies).

Membranes were blocked in TBS-T 5% skim milk for 1 h, incubated overnight with primary antibody and then with secondary antibody for 1 h. Proteins were visualized with Amersham ECL Western Blotting Detection Reagents (GE Healthcare, Amersham, Buckinghamshire, UK) and detected with the Bio-Rad Image Lab system (Bio-Rad, Hercules, California, USA). Primary antibodies used were: anti-VDR (Clone 9A7γ.E10.E4, Thermo Scientific, Rockford, IL, USA), anti-β-catenin, anti-β-actin, (Cell Signaling Technology, Danvers, MA, USA), GSK-3β (BD Biosciences, Franklin Lakes, NJ, USA), and anti-caspase 3 (Merck Millipore, Frenchs Forest, NSW, Australia).

### Animal models

Five-week-old female and male BALB/c nu/nu mice were purchased from the Animal Resources Centre, Canning Vale, WA, Australia. Previously studies in our laboratory confirmed these mice to be fully replete for 25(OH)D levels.^8,10^ Murine xenograft models were employed to study breast (MDA-MB-231, female mice) or prostate (PC3, male mice) cancer cell growth in bone and, orthotopically, in either the mammary fat pads (female mice) or the subcutaneous tissue of the inferior-lateral flank (male mice), as described previously.^6,22^ In accordance with Animal Welfare Guidelines and approved protocols, all mouse manipulations were performed inside a laminar-flow hood under aseptic conditions whilst maintaining general anesthesia following intra-peritoneal injection of ketamine/xylazine (75/10 mg·kg^−1^). The analgesic carprofen (Rimadyl) (5 mg·kg^−1^ subcutaneously) was administered before inoculation to minimize post-surgical pain.

For the orthotopic and subcutaneous models, cells were prepared as above and suspended in a mixture of cold Matrigel and PBS (1:1) at a concentration of 1×10^7^cells per mL. 100 μL of the suspension was either implanted orthotopically into the mammary fad pad or subcutaneously at the flank (*n*=8-10 per group). Tumor growth was measured by calipers every second day from day 6 onwards and tumor volume was determined using the modified ellipsoidal formula [volume=(length×width^2^)/2]. For the intra-tibial xenograft model, 10 μL of cell suspension (for MDA, 5×10^4^ cells per injection, for PC3 cells 10^5^ cells per injection) were slowly injected through the tibial plateau of the left proximal tibia using a Hamilton syringe.^9^ Contralateral tibiae were injected similarly with vehicle alone (PBS). For the monitoring of lytic bone lesions *in vivo*, mice were anesthetized as above and assessed by digital radiography: for MDA cells, images were taken on days 10, 17 and 21 post cell injection; for PC3 cells, images were taken on days 17, 24 and 31 post cell injection. Lytic bone areas within the tibiae were measured using interactive image analysis software (ImageJ, NIH, Bethesda, Maryland, USA) and the size of osteolytic lesions was calculated. The growth of lytic lesions was compared among experimental groups. The intratibial experiments were terminated before mice showed adverse effects of the tumor growth.

### Histological staining and immunohistochemistry

Tibiae were fixed for 36 h in 4% paraformaldehyde buffered with 0.1 mol·L^−1^ phosphate buffer (pH 7.4) and decalcified in 10% EDTA at 4 °C for 2 weeks. The tissues were then processed and embedded in paraffin. Four-micron sections were cut from each specimen and stained with haematoxylin and eosin for routine histological examination. Osteoclasts were detected via histochemical analysis of tartrate-resistant acid phosphatase (TRAcP), using naphthol AS-MX phosphate as substrate and fast red violet Luria-Bertani salt (both from Sigma Chemical, St. Louis, MA, USA) as a stain for the reaction product; incubation was performed at room temperature for 30 min.

Apoptotic cells were identified using terminal deoxynucleotidyl transferase–mediated dUTP nick end labeling (TUNEL) (ApopTaq Peroxidase *In Situ* Apoptosis Detection Kit, Millipore, Temecula, CA, USA). For immunohistochemistry, antigen retrieval was achieved by immersing sections in 10 mmol·L^−1^ citric acid at 70 °C for 90 minutes. The following rabbit polyclonal antibodies and dilutions were used: Ki67 (NeoMarkers, Fremont, CA, USA, 1:200), VDR (Chemicon, AB3786, Temecula, CA, USA, 1:200), and β-catenin (Cell Signaling, 1:500). Signals were detected using biotinylated goat anti-rabbit secondary antibodies (Vectastain ABC-Peroxidase Kits, Vector Laboratories, Burlingame, CA, USA) and DAB (Vector Laboratories). All sections were counterstained with Harris Hematoxylin.

### Micro-computed tomography imaging

After tissue harvest, representative micro-computed tomography (CT) images of tibiae were obtained using a SkyScan 1172 scanner (SkyScan). Scanning was done at 100 kV, 100 μA using a 1-mm aluminum filter. In total, 1 800 projections were collected at a resolution of 6.93 μm per pixel. Reconstruction of sections was done using a modified Feldkamp cone-beam algorithm with beam hardening correction set to 50%. VGStudio MAX software (Volume Graphics GmbH) was used to obtain three-dimensional visualization of tibiae from reconstructed sections.

### Bone histomorphometry

Histomorphometric analysis of the proximal tibial metaphysis was conducted to evaluate bone volume, tumor burden and static measures of bone resorption. Measurements were performed in longitudinal 4 μm thick sections stained with haematoxylin and eosin (×12.5 magnification) using the OsteoMeasure System (Osteometrics, Atlanta, GA, USA). Multiple sagittal sections were cut through each tibial sample and stained. The sagittal section with largest tumor area was selected and the tumor area was measured and used as an index of tumor burden. Cortical bone area was measured in the same sections. Osteoclasts were identified as TRAcP-positive multinucleated cells at the tumor–bone interface. The number of osteoclasts per millimeter of tumor–bone interface was calculated at a magnification of ×200.

### Statistical analysis

All data were presented as the mean±SE. Statistical analyses were conducted using one-way ANOVA followed by Bonferroni’s adjustment where there were multiple comparisons (SPSS 17.0 for Windows, SPSS, Chicago, IL, USA). Significance was accepted at *P*< 0.05.

## Results

### Knockdown of VDR expression in MDA-MB-231 cells

**Figure 1:**
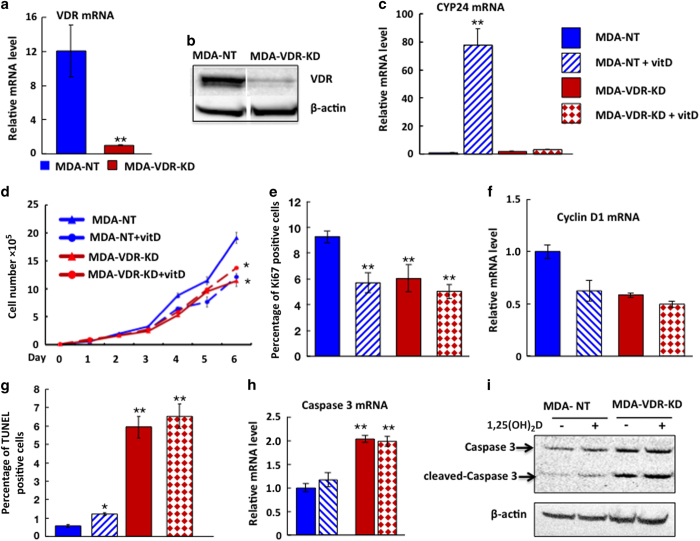
VDR knockdown in MDA-MB-231 cells reduces cell growth *in vitro* in a ligand-independent manner**.** (**a**,**b**) Compared to non-target controls (MDA-NT, cells transfected with non-target RNA), VDR mRNA (**a**) and protein (**b**) expression were knocked down by approximately 80% in MDA-VDR-KD cells at 24 h post plating. **P<0.01*.* (**c**) Treatment of MDA-NT and MDA-VDR-KD cells with 10^−8^ mol·L^−1^ 1,25(OH)_2_D_3_ for 8 h increased CYP24 expression by 80-fold in MDA-NT cells with no appreciable response in MDA-VDR-KD cells. **P<0.01* *compared to baseline. (**d–i**) Culture of MDA-NT and MDA-VDR-KD cells under ligand-free conditions: Compared to NT cells, cell growth (**d**) and cell proliferation (Ki67 immunoreactivity, (**e**) as well as Cyclin D1 mRNA of VDR-KD cells (**f**) were reduced by 40%, 36% and 50%, respectively. However, apoptosis was increased 6-fold (**g**) and Caspase 3 mRNA and protein was increased by 50% (**h,i**). Treatment of MDA-NT cells with 10^−8^ mol·L^−1^ 1,25(OH)_2_D_3_ reduced both cell growth (**d**), Ki67 positivity (**e**) and Cyclin D1 mRNA expression (**f**) and induced a 2-fold increase in cell apoptosis (**g**) as well as Caspase 3 mRNA and protein expression (**h**,**i**) compared to untreated MDA-NT cells. In contrast, the same treatment had no effect on the growth or apoptosis rates of MDA-VDR-KD cells (**d–i**). Asterisks denote significant difference from untreated MDA-NT cells (*P<0.05; **P<0.01). *In vitro* experiments were performed in triplicate and repeated at least three times. Results shown are from a single representative experiment. Data are expressed as mean±s.e.m. (*n*=3)*.*

Relative to shRNA non-target controls (MDA-NT cells transfected with non-target RNA), knockdown efficiency in MDA-VDR-KD cells was ~80% at the mRNA and protein level at 24 h post plating (Figure 1a and b). To assess VDR ablation on a functional basis, MDA-VDR-KD and MDA-NT cells 24 h post plating were treated for 8 h with 10^−8^ mol·L^−1^ 1,25(OH)_2_D_3_ in media supplemented with 2% charcoal stripped heat-inactivated fetal bovine serum and the expression of CYP24, a downstream target gene of the classical VDR signaling pathway, was measured. As expected, treatment with 1,25(OH)_2_D_3_ strongly induced CYP24 expression in MDA-NT cells (>75-fold). In contrast, MDA-VDR-KD cells demonstrated no significant change in CYP24 expression, confirming efficient abrogation of VDR signaling in these cancer cells (Figure 1c).

### VDR knockdown in MDA-MB-231 cells reduces cell proliferation and increases cell apoptosis: *in vitro* studies

As 1,25(OH)_2_D_3_ treatment suppresses cancer cell growth *in vitro*, and vitamin D deficiency enhances breast and prostate cancer growth in animal models of bone metastasis,^8,10,13^ we hypothesized that ablation of the VDR in human breast cancer cells would promote tumor growth. As expected,^25^ treatment of VDR-expressing MDA-NT cells with 10^−^^8^ mol·L^−1^ 1,25(OH)_2_D_3_ significantly reduced *in vitro* cell growth (Figure 1d-f) and induced a 2-fold increase in apoptosis compared to MDA-NT cells (Figure 1g). Surprisingly, however, when cultured under ligand-free conditions the growth of MDA-VDR-KD cells was also significantly reduced, with growth curves similar to those of 1,25(OH)_2_D_3_ treated MDA-NT cells. As expected, treatment of VDR knockdown cells with 1,25(OH)_2_D_3_ had no further effect on growth (Figure 1d). Compared to NT controls, cell proliferation was significantly reduced in MDA-VDR-KD cells, again of similar magnitude to that seen in 1,25(OH)_2_D_3_-treated MDA-NT cells (Figure 1e and f). In contrast, VDR knockdown was associated with a pronounced rise in cell apoptosis: Thus, the proportion of TUNEL-positive cells was 12-fold higher in MDA-VDR-KD cells compared to MDA-NT controls (Figure 1g). In keeping with these observations, Caspase 3 mRNA expression and cleaved Caspase 3 protein levels were significantly higher in MDA-VDR-KD than NT cells (Figure 1h and i).

### VDR knockdown in MDA-MB-231 cells reduces cell growth: *in vivo* studies

In order to examine the effects of VDR knockdown *in vivo,* MDA-VDR-KD and MDA-NT cells were xenografted into vitamin D-replete nude mice either via orthotopic injection into the mammary fat pad or by inoculation into the left tibia.^22^

**Figure 2:**
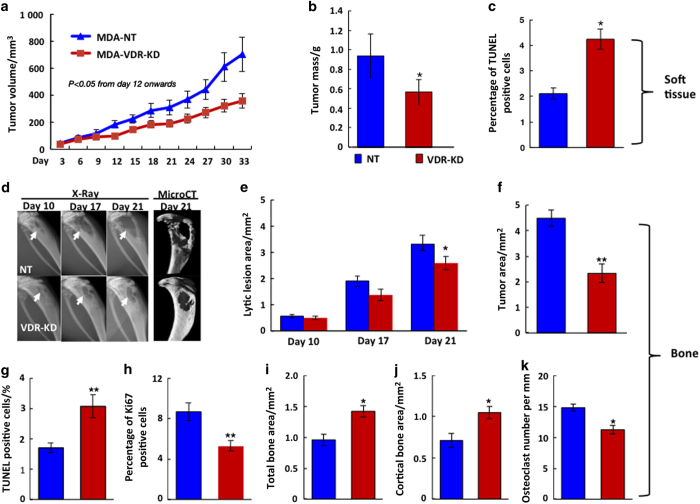
VDR knockdown in MDA-MB-231 cells reduces tumor growth *in vivo. *(**a–c**) Orthotopic implantation: When implanted into the mammary fat pad, tumors derived from MDA-VDR-KD cells grew significantly slower than those induced by MDA-NT cells (*n*=10) (**a**). At study endpoint (day 33 post implantation), tumor weight (**b**) was reduced by 40% while the proportion of apoptotic cancer cells (**c**) was increased by 36% in MDA-VDR-KD compared to MDA-NT tumors (*n*=10). Asterisks denote significant difference from controls (*P<0.05). Data are mean±s.e.m. (**d–j**) Intra-tibial implantation: At endpoint (day 21 post implantation) lytic lesion size on X-ray, micro-CT (**d**,**e**) and histological tumor area (**f**) were significantly smaller in mice implanted with MDA-VDR-KD than with MDA-NT cells (*n*=15). Compared to NT controls, tumors derived from VDR knockdown cells were characterized by an increased proportion of apoptotic cancer cells (**g**) and lower mitotic activity (**h**). At the bone/tumor interface, tumors derived from VDR knockdown cells exhibited increased total and cortical bone area (**i**,**j**), and reduced osteoclast number (**k**). Asterisks denote significant difference from MDA-NT (*P<0.05; **P<0.01). Data are mean±s.e.m*.*

Following orthotopic implantation of MDA-MB-321 cells into the mammary soft tissue, VDR ablation was associated with significantly reduced tumor growth compared to VDR-expressing NT controls (Figure 2a). At endpoint (day 33 post implantation (p.i.)), tumor mass was 40% lower and apoptosis was 36% higher in tumors derived from MDA-VDR-KD cells compared to those grown from MDA-NT cells (*n*=10 per group, *P*<0.05; Figure 2b and c).

When MDA-NT and MDA-VDR-KD cells were implanted into the tibiae, tumors derived from VDR knockdown cells produced significantly smaller lytic lesions (Figure 2d and e) and tumor areas (Figure 2f) at endpoint (day 21 p.i.) than tumors generated by NT cells (*n*=15, *P*<0.05). In keeping with these phenotypes, tumors derived from VDR knockdown cells contained a higher proportion of apoptotic cells (+80%) and demonstrated lower mitotic activity (−39%) (Figure 2g and h). Implantation of MDA-VDR-KD cells also resulted in significantly reduced bone destruction (as demonstrated by significantly greater total and cortical bone area compared to MDA-NT; Figure 2i and j) and reduced osteoclast number at the bone/tumor interface (Figure 2k). These results indicate that knockdown of VDR in breast cancer cells changes tumor growth characteristics *in vivo* regardless of the tissue environment.

### VDR knockdown in PC3 cells—*in* vi*tro* and *in vivo* studies

**Figure 3:**
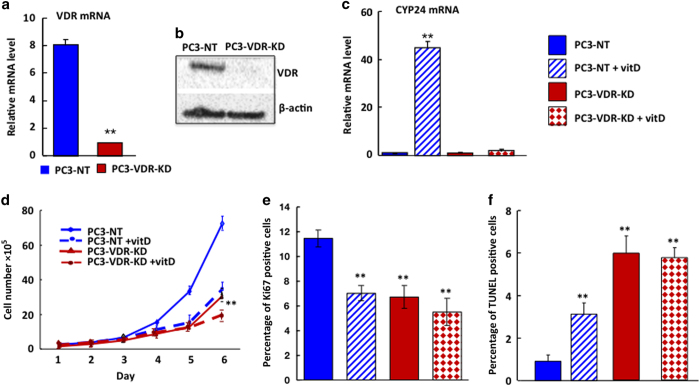
Effects of VDR knockdown in prostate cancer (PC3) cells—*in** **vitro* studies. (**a**,**b**) Compared to non-target controls (PC3-NT, cells transfected with non-target RNA), VDR mRNA (A) and protein (B) expression was knocked down by 80% in PC3-VDR-KD cells at 24 h post plating. **P< 0.001*.* (**c**) Treatment of PC3-NT and PC3-VDR-KD cells with 10^−8^ mol·L^−1^ 1,25(OH)_2_D_3_ for 8 h increased CYP24 expression by more than 40-fold in NT cells with no appreciable response in knockdown cells. **P<0.001 compared to baseline*.* (**d-f**): Culture of PC3-NT and PC3-VDR-KD cells under ligand-free conditions. Compared to NT cells, cell growth (**d**) and cell proliferation (Ki67 immunoreactivity, (**e**) of MDA-VDR-KD cells were reduced by 49% and 41%, respectively, while apoptosis was increased to six fold (**f**). Treatment of PC3-NT cells with 10^−^^8^ mol·L^−1^ 1,25(OH)_2_D_3_ reduced cell growth by 51% (**d**) and Ki67 positivity by 38% (**e**) while inducing a 3-fold increase in apoptosis (**f**) compared to untreated PC3-NT cells. In contrast, the same treatment had no effect on the growth or apoptosis rates of PC3-VDR-KD cells (**d–f**). *P<0.05; **P<0.01 compared to respective controls. *In vitro* experiments were performed in triplicate and repeated at least three times. Results shown are from a single representative experiment. Data are expressed as mean±s.e.m. (*n*=3)*.*

**Figure 4:**
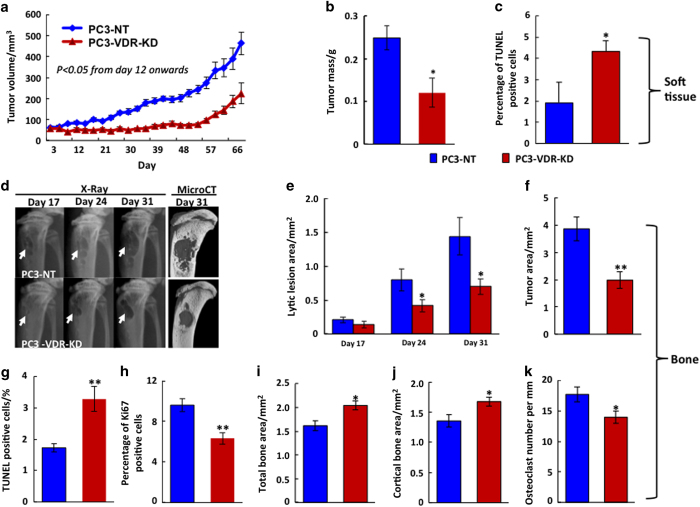
VDR knockdown in PC3 cells reduces tumor growth *in vivo. *(**a–c**)* *Orthotopic implantation (soft tissue, male nude mice). Tumors derived from PC3-VDR-KD cells grew significantly slower than those induced by PC3-NT cells (*n*=9) (**a**). At study endpoint (day 69 p.i.) tumor weight (**b**) was reduced by 40% and the proportion of apoptotic cells (**c**) was increased by 125% in PC3-VDR-KD compared to PC3-NT tumors (*n*=10). Asterisks denote significant difference from controls (*P<0.05). Data are mean±s.e.m. (**d–j**) Intra-tibial implantation: Lytic lesion size (**d**,**e**) and tumor area (**f**) at endpoint (day 31 p. i.) were significantly smaller in mice implanted with PC3-VDR-KD cells compared to those implanted with PC3-NT cells (*n*=12). Compared to NT controls, tumors derived from PC3-VDR-KD cells were characterized by increased apoptosis (**g**) and lower mitotic activity (**h**), and at the bone/tumor interface exhibited increased total and cortical bone area (**i**,**j**) and reduced osteoclast number (**k**). Asterisks denote significance difference from controls (*P<0.05; **P<0.01). Data are mean±s.e.m.

In order to investigate whether the effects of VDR ablation on cancer cell behavior are reproducible across different types of cancer, we next studied the human prostate cancer cell line, PC3, in analogous experiments. Compared to non-target controls (PC3-NT, cells transfected with non-target RNA), VDR mRNA and protein expression was reduced by 80% in PC3 cells at 24 h post plating (PC3-VDR-KD) (Figure 3a and b). Functionally effective ablation of the VDR was again confirmed by a significant reduction in CYP24 expression following stimulation with 10^−8^ mol·L^−1^ 1,25(OH)_2_D_3_ (Figure 3c). The *in vitro* growth curves of PC3 cells exhibited similar results to those seen with MDA-MB-231 cells: Knockdown of VDR expression significantly reduced PC3 cell growth, while at the same time stimulating cancer cell apoptosis. These changes were independent of the effects of 1,25(OH)_2_D_3_ and of similar magnitude to those seen when PC3-NT cells were treated with 1,25(OH)_2_D_3_ (Figure 3d-f). Subsequent *in vivo* studies in vitamin D-replete nude mice revealed growth patterns comparable to those observed in MDA-MB-231 cells: Thus, in both soft tissue and bone, the growth of tumors derived from PC3-VDR-KD cells was significantly reduced when compared to tumors grown from PC3-NT cells (Figure 4a-k). These results suggest that the effects of VDR ablation on cancer cell growth are similar in human breast and prostate cancer cell lines, and may therefore be generic across different cancers.

### Knockdown of VDR in MDA-MB-231 cells reduces the Wnt/β-catenin signaling

**Figure 5:**
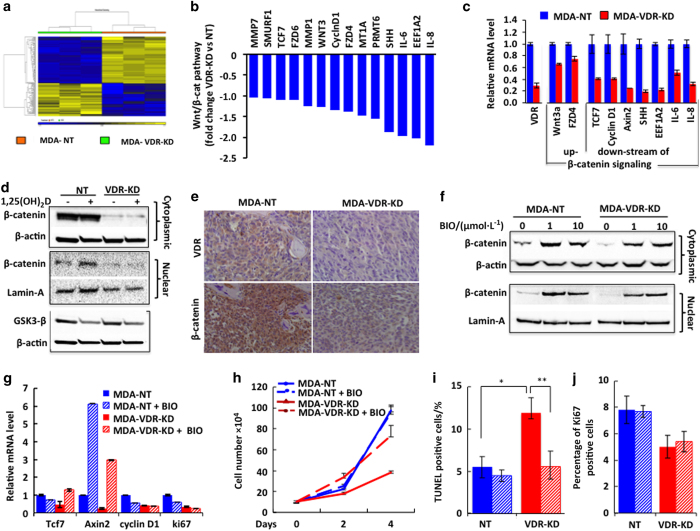
Knockdown of the VDR in MDA-MD-231 breast cancer cells downregulates Wnt/β-catenin signaling pathway. (**a**) Hierarchical clustering of 121 differentially expressed genes (*P*<0.05) between MDA-VDR-KD compared to MDA-NT cell lines (microarray data set) showed distinctive expression patterns. The three lanes represent gene array patterns derived from three separate cultures of the same cell line (MD-NT or MDA-VDR-KD) cultured in identical conditions for the same amount of time. (**b**) From this list, candidate genes belonging to the Wnt/β-catenin pathway, downstream of β-catenin were shown to be downregulated. (**c**) qRT-PCR was used to validate these genes whereby reflecting processes in decreased proliferation and increased apoptosis. (**d**,**e**) Both cytoplasmic and nuclear β-catenin protein levels were reduced in MDA-VDR-KD compared to MDA-NT cells. Treatment with 10^−8^ mol·L^−1^ 1,25(OH)_2_D_3_ induced nuclear β-catenin protein levels in MDA-NT but not in MDA-VDR-KD cells while GSK-3β expression remained unchanged in 1,25(OH)_2_D_3_-treated MDA-NT and MDA-VDR-KD cells (**d**). Reduced β-catenin protein levels in tumors derived from VDR knockdown cells relative to NT controls (IHC stain, **e**). (**f–j**) Inhibition of GSK-3β via treatment of VDR-NT and VDR-KD cells with 1 μmol·L^−1^ BIO resulted in increased expression levels of β-catenin protein (**f**) as well as Axin2, cyclin D1 and Ki67 mRNA (**g**). Inhibition of GSK-3β was associated with increased growth of MDA-VDR-KD cells (**h**), significantly reduced apoptosis (**i**) but unchanged proliferation of VDR-KD cells, as assessed by Ki67 expression (**j**). Asterisks denote significance difference from controls (*P<0.05; **P<0.01). Data are mean±s.e.m.

To further elucidate the function of the VDR in MDA-MB-231 breast cancer cells and the underlying molecular events, we next assayed global gene expression profiles comparing MDA-VDR-KD with MDA-NT cells *in vitro*. Hierarchical clustering of the top 150 differentially expressed genes revealed distinctive gene expression patterns between MDA-VDR-KD and MDA-NT cells (Figure 5a). Pathway enrichment analysis demonstrated that genes within the Wnt/β-catenin pathway were all downregulated in VDR knockdown cells (Figure 5b). Expression of these genes was further validated by real-time RT-PCR, establishing significant downregulation of relevant genes most of which are downstream of β-catenin (Figure 5c). Western blot analysis indicated that both cytoplasmic and nuclear β-catenin protein levels were reduced in MDA-VDR-KD compared to MDA-NT cells. Treatment with 1,25(OH)_2_D_3_ induced nuclear β-catenin protein levels in MDA-NT but not in MDA-VDR-KD cells (Figure 5d). This reduced β-catenin protein levels could be due to, at least in part, the reductions of Wnt3a and FZDR, the upstream initiative genes (Figure 5c) but were not due to GSK-3β as its expression is not altered in MDA-VDR-KD cells (Figure 5d).

*Ex vivo* analyses of tumor tissue confirmed that β-catenin protein levels were reduced in tumors derived from VDR knockdown cells relative to NT controls (Figure 5e). There is solid evidence to indicate that aberrant activation of Wnt/β-catenin signaling inhibits cell apoptosis while promoting cell proliferation.^26–28^ Thus, the slower growth of tumors generated by VDR knockdown cells may be due to a reduction in Wnt/β-catenin activation. To test this hypothesis, we treated the cells with the GSK-3β inhibitor, 6-bromoindirubin-3’–oxime (BIO, Sigma), to stabilize and therefore enhance β-catenin levels.^23^ At concentrations of 1 and 10 μmol·L^−1^, BIO elevated cytoplasmic β-catenin and increased nuclear β-catenin protein levels in both MDA-NT and MDA-VDR-KD cells (Figure 5f). Consequently, Axin2 mRNA levels were increased 6- and 3-fold in BIO-treated NT and VDR-KD cells, respectively (Figure 5g). In contrast, BIO had no effect on mRNA expression of Cyclin D1 or Ki67 (Figure 5g). Growth curve analyses showed that treatment of VDR knockdown cells with BIO restored cell growth to approximately 70% of NT controls (Figure 5h). Consistent with the results for mRNA expression (Figure 5g), immunohistochemistry for Ki67 confirmed that treatment of NT or VDR-KD cells with BIO had no effect on proliferation (Figure 5j). In contrast, treatment of MDA-VDR-KD cells with BIO resulted in a significant reduction in the proportion of apoptotic cells, similar to the levels observed in NT cells (Figure 5i).

## Discussion

Our *in vitro* and *in vivo* studies demonstrate that the VDR has a function in promoting the growth of breast and prostate cancer cells, independent of its cognate ligand, 1,25(OH)_2_D. This previously unrecognized function of the VDR is operational both within and outside the bone microenvironment. Mechanistically, we established that knockdown of the VDR in cancer cells interferes with the Wnt/β-catenin signaling pathway. Thus, VDR knockdown is associated with a robust reduction in cytoplasmic β-catenin protein levels, resulting in enhanced cell apoptosis and reduced proliferation rates. Of note, stabilization of β-catenin and hence re-activation of β-catenin signaling in VDR knockdown cells partly restored cell growth, mostly through inhibition of cell apoptosis.

We previously reported that vitamin D deficiency promotes the growth of human breast and prostate cancer cells in bone.^5,8,10,13^ This acceleration in cancer cell growth was, at least in part, attributable to the effects of vitamin D deficiency on the bone microenvironment, as hypovitaminosis D—via an increase in parathyroid hormone secretion—induces a significant increase in osteoclast-mediated bone resorption.^5,8,10,13^ In addition, treatment with 1,25(OH)_2_D_3_ inhibits breast and prostate cancer cell growth *in vitro* and *in vivo*.^8,10^ This and the fact that inhibition of bone remodeling failed to completely reverse the pro-proliferative effects of vitamin D deficiency provided evidence that vitamin D signaling may be directly involved in the control of tumor growth in bone. Disrupting vitamin D signaling in cancer cells would therefore be expected to promote cell proliferation and tumor growth, similar to the effects of vitamin D deficiency.

However, contrary to this assumption and our expectations, we found that silencing of the VDR in breast and prostate cancer cell lines consistently resulted in reduced cell growth and increased apoptosis *in vitro*. Importantly, these effects were seen not only in the presence but also in the absence of 1,25(OH)_2_D_3_. Furthermore, the effects of VDR knockdown on tumor growth, cancer cell proliferation and apoptosis observed *in vitro* were fully reproducible *in vivo,* evident not only in bone but also in soft tissues, and independent of whether human breast or prostate cancer cell lines were used. The fact that the VDR knockdown cells are insensitive to treatment with 1,25(OH)_2_D_3_ is particularly relevant to the *in vivo* experiments, as these were performed in vitamin D-replete mice, thus providing evidence that the reduction in tumor growth seen *in vivo* with VDR-KD cells must be independent of vitamin D. Taken together, our findings indirectly suggest that the VDR holds hitherto unknown functions that promote cancer cell growth independent of its ligand.

Most of the known biological effects of 1,25(OH)_2_D are mediated through binding to the VDR. Ligand binding causes the VDR to form a heterodimer with the retinoid X receptor and to translocate from the cytoplasm to the nucleus, where the complex binds to vitamin D-responsive elements in the regulatory region of its target genes.^15^ In the absence of vitamin D, the VDR is mostly retained in the cytoplasm,^29,30^ however, recent evidence suggests that shuttling of the unliganded VDR to the nucleus produces basal transcriptional activity via binding to co-repressor or co-activators.^30–35^ Global chromatin immunoprecipitation (ChIP) analyses have also indicated that a significant number of DNA-binding sites are occupied by the VDR in the absence of its cognate ligand.^36^

There is little data available regarding a potential unliganded function of the VDR. Previous studies in skin suggest that the unliganded VDR plays a role in the context of normal keratinocyte stem cell function (through interaction with the canonical Wnt pathway).^20,37,38^ The fact that ablation of VDR expression in cancer cells results in a marked inhibition of cell and tumor growth raises the possibility of the *unliganded* VDR being involved in the regulation of cancer cell proliferation and apoptosis. However we acknowledge that this conclusion cannot be seen as definitive due to incomplete VDR ablation. Our data indicate that the pro-apoptotic and anti-proliferative effects of VDR knockdown are mediated through inhibition of the Wnt/β-catenin signaling pathway. Indeed, stabilizing β-catenin in VDR-KD cells partially restored the growth of these cancer cells. In this context, it was interesting to note that *in vitro*, BIO-induced stabilization of β-catenin reversed the effect of VDR-KD on cell apoptosis but not on cell proliferation (for example, Ki67, cyclin D1 expression). Also, compared to NT controls, the expression of interleukin-6 (IL-6) and IL-8 mRNA was significantly reduced in breast cancer cells with disrupted VDR signaling. We have previously shown that IL-6 mediates direct paracrine–autocrine signaling between cells of the osteoblast lineage and cancer cells, thereby enhancing the growth of breast cancers in bone.^22^ The reduced growth of VDR knockdown cells and tumors derived from such cells may, at least in part, be due to their diminished expression and secretion of interleukins like IL-6.

Wnt/β-catenin signaling plays a crucial role in breast and prostate cancer cell growth.^26–28^ Aberrant activation of the Wnt/β-catenin signaling pathway in cancer cells is associated with the modification of pivotal processes such as Wnt expression, β-catenin stabilization or degradation, and nuclear translocation of β-catenin. In the nucleus, β-catenin complexes with T-cell factor and facilitates transcription of target genes activating cell proliferation and inhibiting apoptosis. Currently, it remains to be seen how the VDR interacts with β-catenin in cancer cells. For example, we did not detect changes in GSK-3β protein levels, suggesting that the low β-catenin levels observed in VDR knockdown cells may not be due to its degradation. Instead, in cells with disrupted VDR signaling we found significant changes in the expression of genes upstream of β-catenin (for example, Wnt3a, FZD4), although it appears that these changes would not fully explain the profound reduction in β-catenin concentrations and signaling.

Clinical research into the role of vitamin D and VDR status in breast and prostate cancer has remained controversial. While it appears that the VDR is present in differentiated breast cancers,^39,40^ the associations between VDR expression, its downstream signaling and patient prognosis or survival still require further clarification.^16,18,41,42^ VDR expression levels may be associated with the transition from benign to invasive breast tumor.^40,43,44^ Some studies showed cancer progression in breast tumors was characterized by reduced CYP27B1 and increased CYP24A1 expression, indicating that during the process of malignant transformation the reduction in VDR expression and higher CYP24A1 levels renders the tumor less sensitive to vitamin D.^40,43^ Our results may help to explain some of the discrepancies between vitamin D status and clinical outcome^45,46^ as tumor behaviour and patient outcome may likely depend on both vitamin D and VDR status. Thus, in the case of vitamin D deficiency, cancer cells may lose the ligand-mediated nuclear signaling effects of the VDR and as a consequence, the unliganded VDR may become the dominant functional form. Hence, in a ligand-depleted context (that is, vitamin D deficiency), the unliganded VDR may be responsible for increased breast cancer cell growth. In the presence of adequate vitamin D, increased nuclear VDR localization and reduced unliganded VDR actions could together reduce breast cancer proliferation. It is possible that local production of 1,25(OH)_2_D, dependent on adequate serum 25(OH)D levels and tissue CYP27B1 activity, may be more important than levels of serum 1,25(OH)_2_D which do not correlate closely with vitamin D status.^47^ These possibilities need to be further investigated by measuring nuclear and unliganded VDR expression in human tumors at different stages of progression and correlating these results with the patient’s vitamin D status in a prospective clinical trial.^46^

In conclusion, the current study demonstrates that the unliganded VDR possesses intrinsic functions that control cancer cell growth in a ligand-independent manner through interference with the Wnt/β-catenin signaling pathway. Targeting the unliganded VDR may open up new avenues for the development of novel diagnostic and therapeutic approaches in breast and prostate cancer.

## Figures and Tables

**Table 1:** Primers used in this article

Primers	Sequence (5′–3′)
hGAPDH	F: TATGACAACGAATTTGGCTACAGR: TGATGGTACATGACAAGGTGC
hVDR	F: ACCTGGACAACAAGAGCGA R: CTCCTTCCTTCTCCTTCTGATG
hCYP24	F: GCATCTTCCATTTGGCGT R: AATACCACCATCTGAGGCGT
Cyclin D1	F: AGAACACGGCTCACGCTTA R: ATCCAGGACTTGTGCCCTT
Caspase 3	F: CAGCACCTGGTTATTATTCTTGG R: TGTCGGCATACTGTTTCAGC
Wnt3a	F: CTCTCCCTCTCTCTCATCTTACATTT R: GCCTCAGGTCTGTTCCTATCA
FZD4	F: ATTCCCACCACAGAACGAC R: CATAGCCACACTTGAGCACAC
TCF7	F: CTGCCCAGGTGACTGACTAAT R: GATTGAAGGCGGAGTAGACG
Axin2	F: AGACGGTGCTTACCTGTTCC R: GCTGCTTGGAGACAATGCT
hIL-6	F: GCATTCCTTCTTCTGGTCAG R: GCCATTTATTTGAGGTAAGCC
hIL-8	F: AGACAGCAGAGCACACAAGC R: CACTGGCATCTTCACTGATTCT
SHH	F: CAGAAACTCCGAGCGATTT R: GCCAAAGCGTTCAACTTGT
EEF1A2	F: CACATCAACATCGTGGTCA R: CGAACTTCTCAATGGTCCTTT
Ki67	F: ACGTCGTGTCTCAAGATCTAGC R: AACGGCTCACTAATTTAACGC
